# Nitric Oxide and Decreases in Resistance Exercise Blood Pressure With Aerobic Exercise Training in Older Individuals

**DOI:** 10.3389/fphys.2019.01204

**Published:** 2019-09-20

**Authors:** Takeshi Otsuki, Fumiko Nakamura, Asako Zempo-Miyaki

**Affiliations:** Faculty of Sport and Health Sciences, Ryutsu Keizai University, Ryugasaki, Japan

**Keywords:** nitric oxide, systolic blood pressure, resistance exercise, aerobic exercise training, arterial stiffness

## Abstract

An exaggerated blood pressure response to resistance exercise is a marker of masked hypertension and a risk factor for future essential hypertension. Habitual aerobic exercise decreases systolic blood pressure (SBP) during resistance exercise in older individuals, but the underlying mechanisms have not been explored. This study tested the hypothesis that nitric oxide (NO) mediates a reduction of resistance exercise SBP with aerobic training in older individuals. Normotensive older adults participated in a 6-week program as a part of the aerobic training group (*n* = 23, exercised for an average of 4.4 d/wk and 59 min/d) or the control group (*n* = 26, asked not to modify their lifestyle during the experimental period). The aerobic exercise intervention increased plasma concentrations of nitrite/nitrate (NOx, end products of NO) and decreased SBP during a one-hand arm curl exercise at 20% and 40% of one-repetition maximum and brachial-ankle pulse wave velocity (an index of arterial stiffness). In the control group, there were no differences in these measures before and after the experimental period. Changes in plasma NOx concentrations during the study period were correlated with changes in resistance exercise SBP. Stepwise regression revealed that changes in plasma NOx concentrations during the experimental period are a significant factor of changes in resistance exercise SBP, independent of age, sex, and changes in serum lipid profile, maximal oxygen uptake, resting SBP, and other variables. These results suggest that NO is associated with decreases in resistance exercise SBP with aerobic training in older individuals and help us better understand why habitual aerobic exercise prevents cardiovascular disease.

## Introduction

An exaggerated blood pressure response to exercise is a risk factor for future cardiovascular disease (Keller et al., 2017; Schultz et al., 2017). To attenuate the blood pressure response, researchers have been investigating the relationships between blood pressure response and lifestyle components such as dietary habits (Michishita et al., 2019) and supplementation (Kim et al., 2018), habitual exercise (Kokkinos et al., 1997; Pitsavos et al., 2011), and medications (Nashar et al., 2004; Chant et al., 2018; Piche et al., 2018), because ischemic heart disease and stroke have been the leading causes of death around the world for the last 15 years. They have resulted in 15.2 million deaths in 2016, according to the World Health Organization [WHO] (2018). Although aerobic exercise, such as treadmill walking or ergometer cycling, has been used as part of exercise testing in these studies (Kokkinos et al., 1997; Nashar et al., 2004; Pitsavos et al., 2011; Chant et al., 2018; Kim et al., 2018; Piche et al., 2018; Michishita et al., 2019), resistance exercise, such as lifting objects and mopping, is part of the activities of daily living. Elevated blood pressure during resistance exercise is a marker of masked hypertension (Koletsos et al., 2019), independent factor of arterial stiffness (Koletsos et al., 2019; Otsuki and Kotato, 2019), and risk factor for future essential hypertension (Chaney and Eyman, 1988). Therefore, it is important to explore lifestyle modifications to decrease blood pressure during resistance exercise.

Kruse et al. (2018b) recently reported that 8 weeks of sodium nitrate supplementation decreased mean blood pressure during resistance exercise in patients with peripheral artery disease. In healthy middle-aged and older individuals, we previously reported that 6 weeks of aerobic exercise training decreases systolic blood pressure (SBP) during resistance exercise (Otsuki et al., 2016). However, the mechanisms underlying the reduction in resistance exercise SBP with aerobic training remain unclear. One potential mechanism is nitric oxide (NO), a vasodilatory factor. In animal studies, blunting of sympathetic vasoconstriction during exercise (functional sympatholysis) was enhanced through a NO-dependent mechanism (Jendzjowsky and Delorey, 2013; Mizuno et al., 2014). Human studies have reported that aerobic training increases plasma concentrations of nitrite/nitrate (NOx, end products of NO) (Maeda et al., 2004; Fujie et al., 2014, 2015) and improves functional sympatholysis (Mortensen et al., 2014). In addition, decreases in resistance exercise SBP by aerobic training are correlated with decreases in arterial stiffness (Otsuki et al., 2016); NO is a potent regulator of arterial stiffness (Wilkinson et al., 2002; Sugawara et al., 2007).

We hypothesized that an increase in NO production resulting from aerobic training decreases SBP during resistance exercise in older individuals. To test this hypothesis, we measured blood pressure during a one-hand arm curl exercise at 20% and 40% of one-repetition maximum (1RM) (Otsuki et al., 2016), because various upper-arm activities at low intensity are needed during activities of daily living, plasma NOx concentrations, and brachial–ankle pulse wave velocity (baPWV, an index of arterial stiffness) before and after a 6-week aerobic training program in older individuals. The aerobic exercise intervention was performed according to our previous study (Otsuki et al., 2016) to elucidate the mechanisms underlying the reduction in resistance exercise SBP with aerobic training demonstrated in the previous study.

## Materials and Methods

### Participants

We recruited participants without any disorders for which exercise is contraindicated (e.g., unstable ischemia and acute low back pain) through community magazines. Participants chose the control (11 men and 15 women, 65 ± 7 years of age [mean ± SD]) or training (9 men and 14 women, 67 ± 8 years) group and volunteered to participate in this study. Participants with treated or untreated hypertension (SBP/diastolic blood pressure [DBP] ≥140/90 mmHg) were excluded (SBP/DBP [mean ± SD]; control group, 110 ± 13/65 ± 8 mmHg and training group, 109 ± 13/64 ± 8 mmHg). In addition, participants with diabetes, on hormone replacement therapy, or who smoked tobacco were excluded. Subjects refrained from alcohol consumption and intense physical activity starting on the day before testing and caffeine consumption on the day of testing. In addition, subjects were instructed not to take NOx from dietary sources (Wang et al., 1997; Himeno et al., 2003) on the day before blood sampling after overnight fasting. Subjects were asked not to change their lifestyle during the experimental period except for participation in this study. Compliance with the study protocol was checked through questions asked before measurements and blood sampling.

All measurements were conducted in an air-conditioned room (air temperature, 25°C). First, resting blood pressure and baPWV were measured after at least 15 min of rest. Second, a muscle stretching and 1RM assessment were performed. Third, resistance exercise test was conducted after at least 5 min of rest following 1RM assessment. Finally, oxygen uptake was monitored during incremental cycling to estimate maximal oxygen uptake.

A power calculation was performed to determine whether stepwise regression can identify independent factors of resistance exercise SBP using G^∗^Power 3, a statistical power analysis program (Faul et al., 2007). The sample size of this study was sufficient when the effect size (f^2^) was ≥0.17, which is slightly greater than a medium (0.15) but less than a large (0.35) effect size (Cohen, 1992).

This study was approved by the Ethics Committee of Ryutsu Keizai University (Approval Number 7) and conformed to the principles of the Helsinki Declaration. All participants gave their written informed consent prior to study participation.

### Resistance Exercise Testing Protocol

Subjects performed a one-hand arm curl exercise using an arm curl bench and dumbbells (Otsuki et al., 2016). At first, they performed two sets of 10 repetitions of the exercise at 20% 1RM. Each repetition was performed for 8 s (3 s of concentric contraction, 1 s of maintaining full flexion, 3 s of eccentric contraction, and 1 s of maintaining a slightly flexed position). The inter-set recovery period was 100 s. Subjects were instructed to continue breathing normally during the exercise. After completion of the 20% 1RM session, 160 s elapsed before the 40% 1RM session commenced in a similar fashion. During exercise testing after the intervention, post-intervention 1RM was used to determine exercise intensity.

### Blood Pressure and Heart Rate Measurement

Blood pressure was measured using oscillometry (DINAMAP; GE Healthcare, Buckinghamshire, United Kingdom) (Otsuki et al., 2016). Resting blood pressure was measured in triplicate after at least 15 min of rest. During resistance exercise, measurements were taken once per set. Exercises were performed with the dominant arm and blood pressure was measured in the non-dominant arm. Heart rate (HR) was calculated using a three-lead ECG (LRR-03; GMS, Tokyo, Japan) (Otsuki et al., 2016). The mean value of each parameter over two sets at 20% and 40% 1RM, respectively, was calculated.

### Blood Chemical Analysis

Blood samples were collected after an overnight fast. Subjects were instructed not to take NOx from dietary sources (Wang et al., 1997; Himeno et al., 2003) on the day before sampling. Plasma NOx concentrations were determined using the Griess method (Green et al., 1982; Otsuki et al., 2015b). The lipid profile and levels of glucose, insulin, and hemoglobin A1c were determined using standard techniques (Otsuki et al., 2015a).

### Arterial Stiffness Assessment

Brachial and post-tibial artery pulse waves were obtained in triplicate in the supine position (BP-203RPE II; Fukuda Colin, Tokyo, Japan) (Otsuki et al., 2016). The device calculated the distance traveled by the pulse waves based on each subject’s height and automatically determined the pulse wave transit time. BaPWV was calculated as the distance divided by the transit time.

### Maximal Oxygen Uptake Estimation

Three-lead ECG (LRR-03; GMS) and breath-by-breath oxygen uptake (AE300S; Minato Medical Science, Osaka, Japan) were monitored during incremental cycling (4 min at 30 W, with a 20 W increase for males or a 15 W increase for females every 2 min to 85% of age-predicted maximum HR) (Otsuki et al., 2016). Maximal oxygen uptake was calculated as oxygen uptake corresponding to the maximum HR using the linear regression line between HR and oxygen uptake.

### 1RM Assessment

Subjects performed a single repetition of the one-hand arm curl exercise using their dominant arm with progressively heavier weights. The heaviest weight that a subject could lift once through a complete range of movement was considered his or her individual 1RM (Otsuki et al., 2016).

### Body Mass Index Measurement

Body weight was measured after an overnight fast using a digital scale (InBody 430; InBody, Seoul, Korea). Height was measured using a digital stadiometer (AD-6227; A & D, Tokyo, Japan). Body mass index (BMI) was calculated as weight divided by square of height.

### Exercise Intervention

Participants in the training group underwent supervised walking (35–50 min) once per week for 6 weeks. HR was monitored during walking using a HR monitor (RS-400; Polar, Kempele, Finland). During the first 10 min of walking, they walked at normal speed as a warm up. Ten minutes after the onset of walking, participants increased their walking speed to the target intensity. Initially, the target intensity was relatively low (60–65% of age-predicted maximal HR). As their exercise tolerance improved, the target intensity was increased to higher level (75% of age-predicted maximal HR). In addition, subjects walked 2–4 times a week on their own at the same pace as during supervised walking and recorded the duration of their walks. Subjects in the control group were asked not to modify their lifestyle during the experimental period.

### Statistical Analysis

**FIGURE 1:**
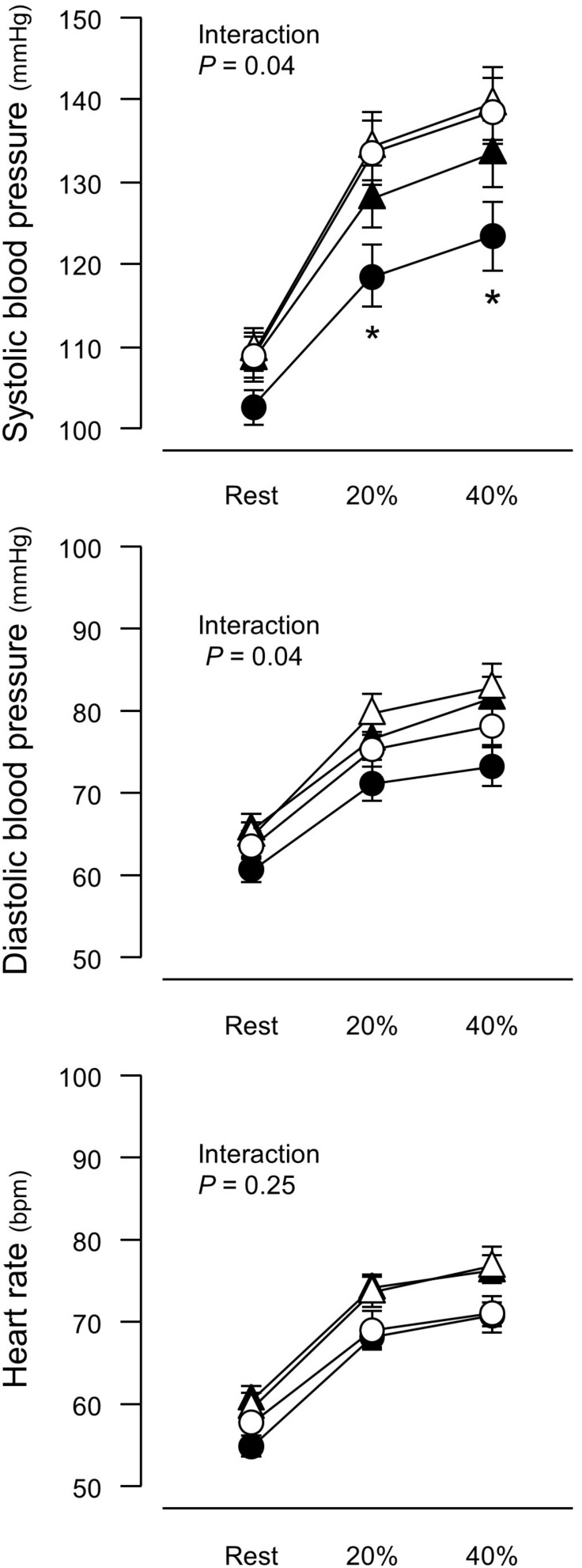
Blood pressure and heart rate before and after a 6-week aerobic exercise intervention period. Values are means (SEs). ^∗^*P* < 0.05 vs. before intervention in the same group. Open symbols, before intervention; closed symbols, after intervention; triangles, control group; circles, training group. 20%, an arm curl exercise at 20% of the one-repetition maximum (1RM); 40%, the exercise at 40% 1RM.

**FIGURE 2:**
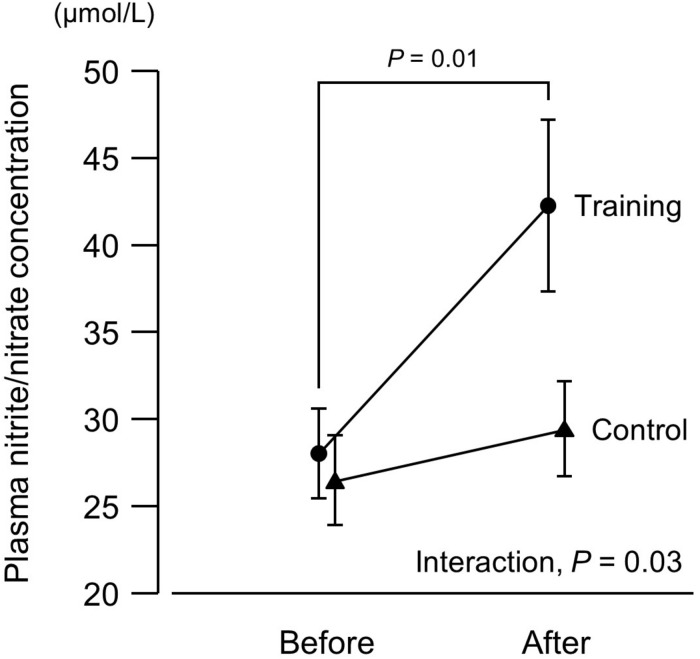
Plasma concentrations of nitrite/nitrate (end products of nitric oxide) before and after a 6-week aerobic exercise intervention period. Values are means (SEs).

**FIGURE 3:**
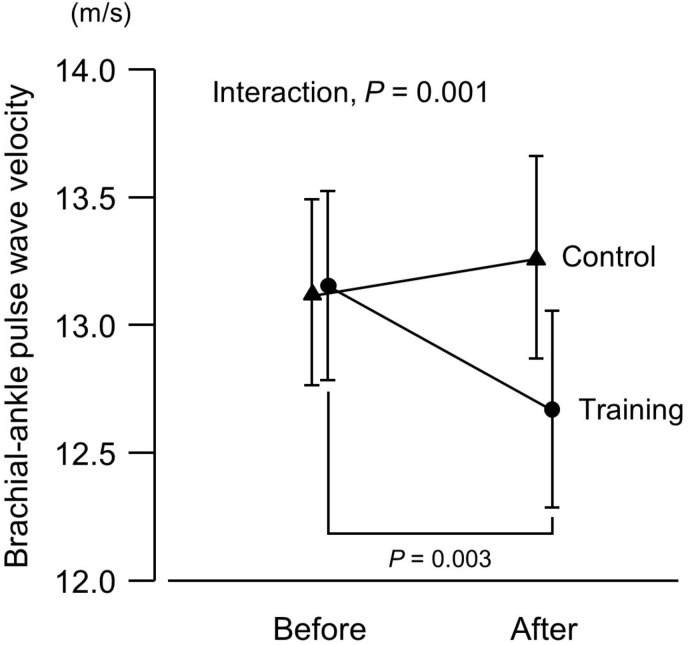
Brachial–ankle pulse wave velocity (an index of arterial stiffness) before and after a 6-week aerobic exercise intervention period. Values are means (SEs).

Characteristics of the study participants (Table 1) are expressed as means (SDs). In comparisons before and after the experimental period (Figures 1–3), values are shown as means (SEs). The unpaired *t*-test was used to detect intergroup differences before the study period. Effects of the intervention were tested using repeated measures two-way analysis of variance (ANOVA). If a significant *F* value was found, a Fisher’s *post hoc* test was performed. Relationships between two variables were investigated using Pearson’s correlation coefficients and partial correlation analysis. Based on a previous study that investigated the relationship between resistance exercise blood pressure and arterial stiffness (Koletsos et al., 2019), the partial correlation analysis adjusted for age and BMI. Stepwise regression was used to identify independent factors of resistance exercise SBP. *P* values <0.05 were considered statistically significant. StatView statistical software (SAS Institute, Cary, NC) was used for analysis.

**TABLE 1:** Characteristics of study participants before and after a 6-week aerobic exercise intervention period.

		**Before**	**After**
*n* (male/female)	Control	11/15	
	Training	9/14	
Age, years	Control	65 (7)	
	Training	67 (8)	
Body mass index, kg/m^2^	Control	22 (2)	22 (2)
	Training	22 (2)	22 (2)
HDL cholesterol, mg/dL	Control	75 (21)	77 (19)
	Training	66 (13)	68 (15)
LDL cholesterol, mg/dL	Control	136 (26)	139 (26)
	Training	135 (28)	131 (25)
Triglycerides, mg/dL	Control	94 (35)	89 (34)
	Training	96 (64)	94 (46)
Glucose, mg/dL	Control	93 (10)	97(10)^∗∗^
	Training	95 (7)	95 (6)
Hemoglobin A1c, %	Control	5.5 (0.3)	5.5 (0.3)
	Training	5.6 (0.4)	5.5 (0.3)
Insulin, μU/mL	Control	4.7 (2.4)	5.1 (2.5)
	Training	5.4 (1.7)	6.7 (2.2)
Maximal oxygen uptake, mL/kg/min	Control	27.2 (5.2)	27.1 (4.9)
	Training	25.0 (5.2)	27.3(5.9)^***^
One-repetition maximum, kg	Control	6.5 (2.5)	6.5 (2.6)
	Training	5.6 (1.9)	5.6 (1.9)

*Values are means (SDs). ^∗∗^*P* < 0.005 and ^∗∗∗^*P* < 0.0005 vs. before intervention. HDL, high-density lipoprotein and LDL, low-density lipoprotein.*

## Results

There were no intergroup differences in the male-to-female ratio, age, BMI, laboratory values, or exercise parameters before the experimental period (Table 1). Subjects in the training group exercised for an average ± SD (range) of 4.4 ± 1.3 (2.3–7.0) d/wk and 59 ± 20 (33–110) min/d at 71 ± 8 (59–91)% of maximal HR and at an 12 ± 1 (9–14) rating of perceived exertion (Borg’s 6–20 scale). Maximal oxygen uptake increased in the training group after the intervention but did not change in the control group. The other variables did not change during the experimental period in both groups, except that plasma glucose concentrations increased in the control group.

There was no intergroup difference in blood pressure or HR before the intervention (Figure 1). In the training group, SBP at 20% and 40% 1RM was lower after the intervention compared to before the intervention. There were no SBP differences in the control group before versus after the experimental period. Resting SBP did not change during the experimental period in both the training and control groups. Although ANOVA demonstrated that trends in DBP changes during exercise were affected by exercise training, there were no significant changes with the intervention in multiple comparisons. Aerobic training increased plasma NOx concentrations (Figure 2) and decreased baPWV (Figure 3) in the training group but there were no changes in the control group.

**FIGURE 4:**
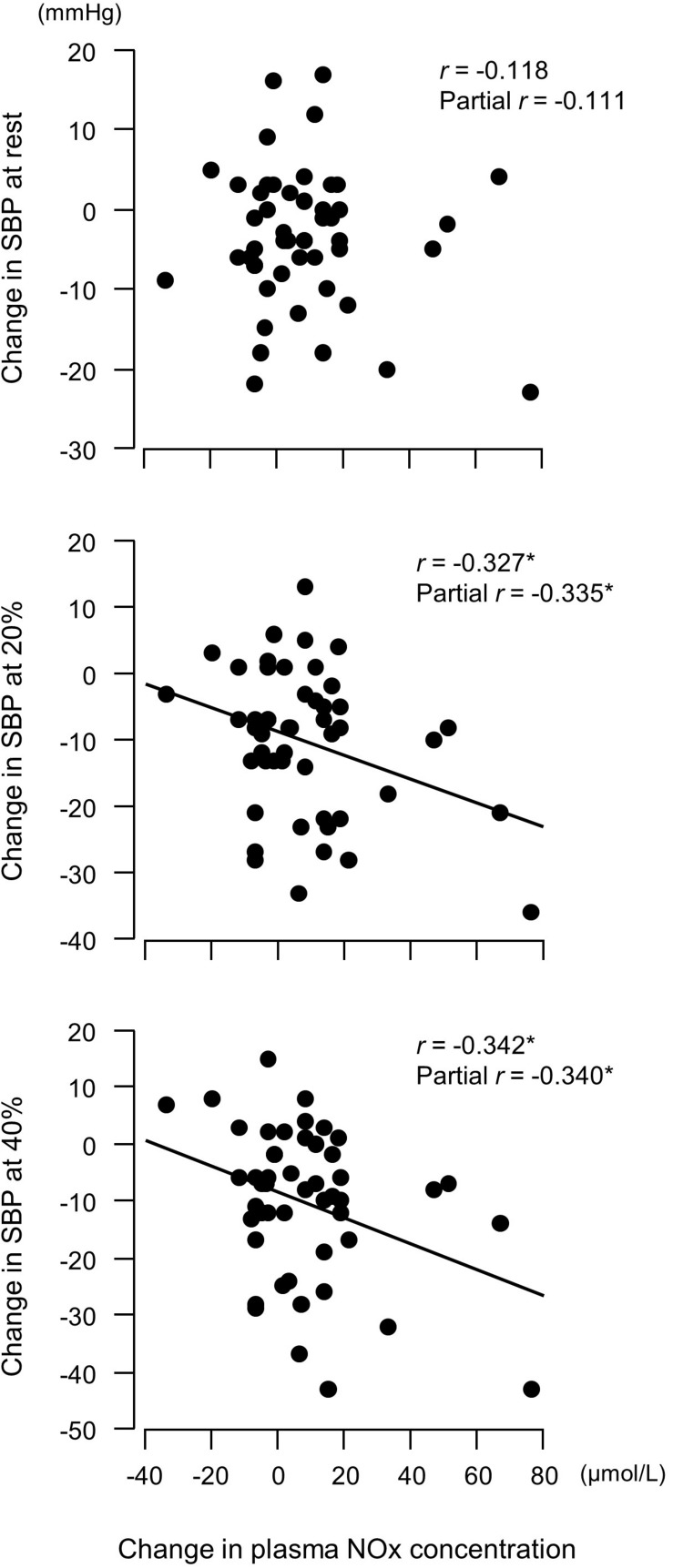
Relationship between changes in plasma concentrations of nitrite/nitrate (NOx, end products of nitric oxide) before versus after a 6-week aerobic exercise intervention period and changes in systolic blood pressure (SBP). Partial correlation analysis adjusted for age and body mass index. ^∗^*P* < 0.05. 20%, an arm curl exercise at 20% of the one-repetition maximum (1RM); 40%, the exercise at 40% 1RM.

**FIGURE 5:**
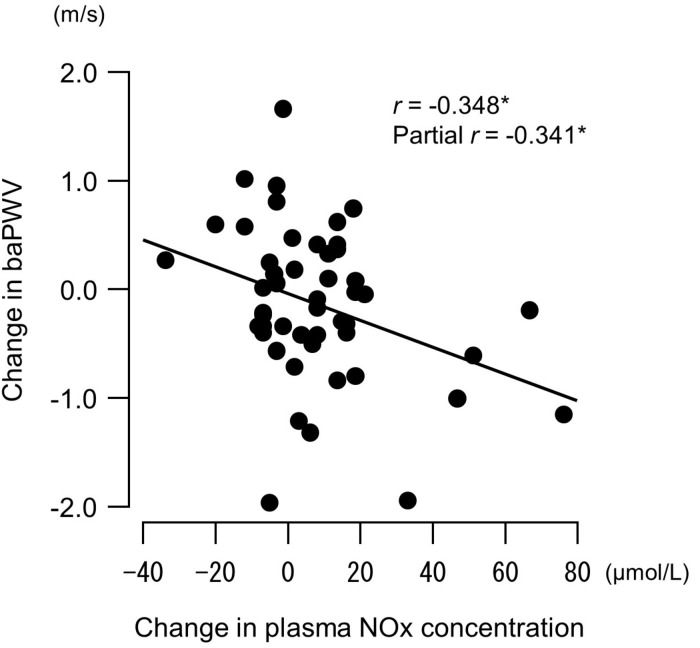
Relationship between changes in plasma concentrations of nitrite/nitrate (NOx, end products of nitric oxide) before versus after a 6-week aerobic exercise intervention period and changes in brachial–ankle pulse wave velocity (baPWV, an index of arterial stiffness). Partial correlation analysis adjusted for age and body mass index. ^∗^*P* < 0.05.

**FIGURE 6:**
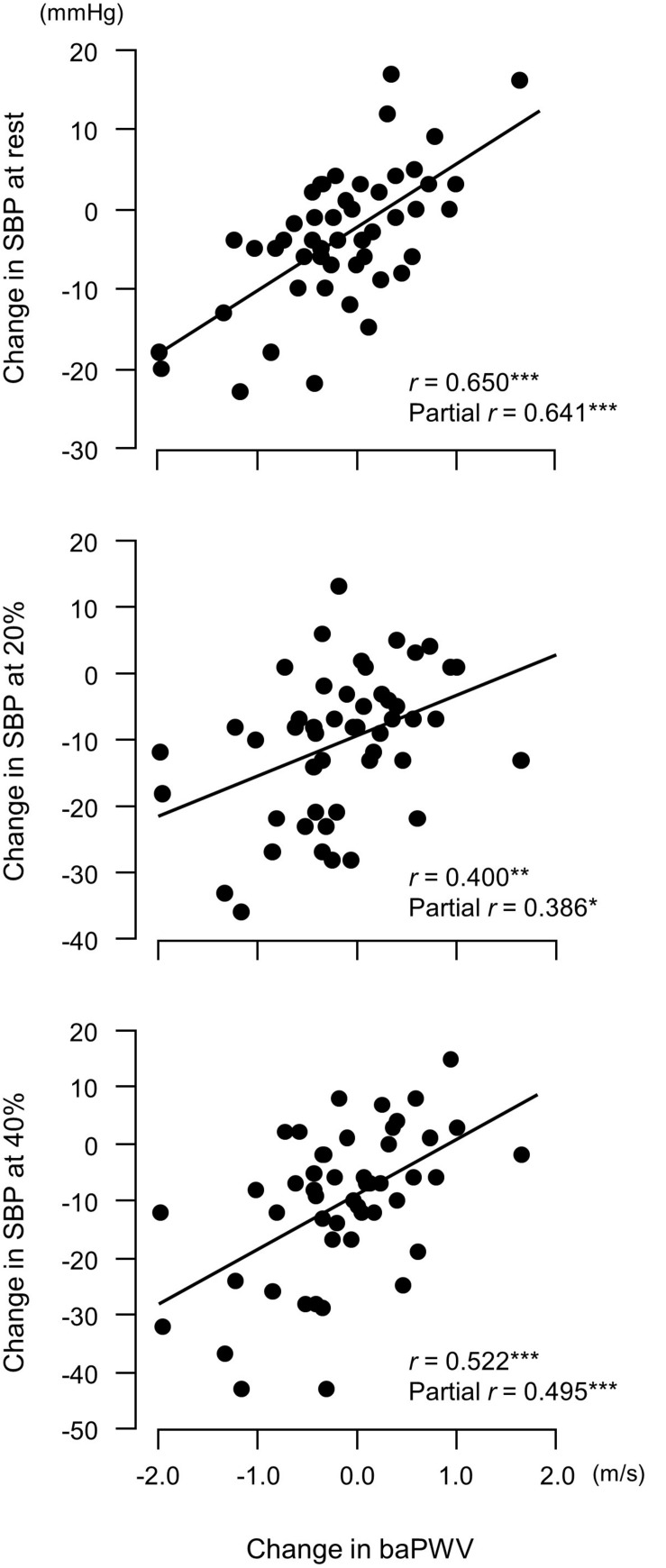
Relationship between changes in brachial–ankle pulse wave velocity (baPWV, an index of arterial stiffness) before versus after a 6-week aerobic exercise intervention period and changes in systolic blood pressure (SBP). Partial correlation analysis adjusted for age and body mass index.^∗^*P* < 0.05; ^∗∗^*P* < 0.005; ^∗∗∗^*P* < 0.0005. 20%, an arm curl exercise at 20% of the one-repetition maximum (1RM); 40%, the exercise at 40% 1RM.

Changes in plasma NOx concentrations before versus after the experimental period were correlated with changes in resistance exercise SBP (Figure 4) and baPWV (Figure 5). In addition, changes in baPWV during the study period were correlated with changes in resting and exercise SBP (Figure 6). In the stepwise regression analysis (Table 2), changes in plasma NOx concentrations during the experimental period and changes in resting SBP were significant factors of changes in resistance exercise SBP. Age; sex; and changes in BMI, laboratory values, and exercise parameters listed in Table 1; resting DBP and HR; and baPWV were not significant factors. When the changes in baPWV during the experimental period were included as a dependent variable (*R*^2^ = 0.537, *P* < 0.0001), only changes in plasma NOx concentrations (regression coefficient = −0.95, SE = 0.36, β = −0.27, *P* = 0.01) and resting DBP (9.93, 1.55, 0.65, and <0.001, respectively) were identified as significant factors.

**TABLE 2:** Stepwise regression analysis to identify independent factors of changes in systolic blood pressure during resistance exercise before versus after a 6-week aerobic exercise intervention period.

		**Changes in exercise SBP**	
		
		**20%**	**40%**
	*R*^2^	0.347	0.442
	*P*	< 0.0001	< 0.0001
Change in plasma NOx concentration	RC	–0.15	–0.18
	SE	0.07	0.07
	β	–0.27	–0.28
	*P*	0.03	0.02
Change in resting SBP	RC	0.62	0.87
	SE	0.15	0.17
	β	0.49	0.57
	*P*	< 0.001	< 0.001
Excluded variables (*P* ≥ 0.05)		Age, sex, and changes in BMI, laboratory and exercise parameters in the Table 1, resting DBP and HR, and baPWV.	

*SBP and DBP, systolic and diastolic blood pressure, respectively; 20%, an arm curl exercise at 20% of the one-repetition maximum (1RM); 40%, the exercise at 40% 1RM; NOx, nitrite/nitrate; RC, regression coefficient; BMI, body mass index; HR, heart rate; and baPWV, brachial-ankle pulse wave velocity.*

## Discussion

The major results of this study are that a 6-week aerobic exercise training program increased plasma NOx concentrations and decreased SBP during resistance exercise at 20% and 40% 1RM and baPWV in older normotensive individuals, whereas there were no changes in these measures in the control group. Of note, this study demonstrated for the first time that increases in plasma NOx concentrations with an exercise intervention are independently associated with decreases in resistance exercise SBP. Decreases in baPWV during the experimental period were also correlated with decreases in resistance exercise SBP. These results support our hypothesis that an increase in NO production with aerobic training decreases SBP during resistance exercise in older individuals.

In this study, aerobic exercise training increased plasma NOx concentrations, which was consistent with previous studies (Maeda et al., 2004; Fujie et al., 2014, 2015). The observed decreases in resistance exercise SBP with aerobic training are also in agreement with a previous study (Otsuki et al., 2016). However, this is the first study to demonstrate that increases in plasma NOx concentrations with aerobic training are correlated with decreases in resistance exercise SBP. The mechanisms through which plasma NOx concentrations are associated with resistance exercise SBP are unclear, but we propose some possibilities. Sympathetic vasoconstriction in contracting muscles is attenuated by local factors such as NO. This phenomenon is termed functional sympatholysis. Aging decreases NO bioavailability and exaggerates blood pressure responses to sympathetic nervous activity (Koch et al., 2003; Dinenno et al., 2005). However, an increase in NO bioavailability through habitual exercise attenuates the blood pressure response to sympathetic stimuli; functional sympatholysis is greater in physically active individuals compared to sedentary peers (Mortensen et al., 2012; Kruse et al., 2018a) and improves with aerobic training in middle-aged adults (Mortensen et al., 2014). Although functional sympatholysis was not measured in this study, the frequency (4.4 d/wk) and duration (59 min/d) of exercise were comparable to those in a previous study that reported an improvement in functional sympatholysis with aerobic training (3–4 d/wk and 60 min/d, respectively) (Mortensen et al., 2014). Vasodilation mediated by NO in contracting muscles might be a mechanism through which increases in NOx concentrations lead to decreases in resistance exercise SBP.

Arterial stiffness may also play a role in the relationship between plasma NOx concentrations and resistance exercise SBP. First, NO bioavailability is a potent regulator of arterial stiffness (Wilkinson et al., 2002; Sugawara et al., 2007). In this study, an increase in plasma NOx concentrations with aerobic training was an independent factor of decreases in baPWV. Second, arterial stiffness and blood pressure interact with each other; elevations in blood pressure increase arterial stiffness, but arterial stiffening elevates blood pressure as well. In particular, the contribution of arterial stiffness to arterial load is more important during exercise than at rest (Otsuki et al., 2006, 2008). Indeed, decreases in baPWV were correlated with decreases in resistance exercise SBP, although decreased baPWV was not a significant factor of decreases in exercise SBP in the stepwise regression. This might be due to multicollinearity between baPWV and resting blood pressure. In previous cross-sectional studies, arterial stiffness was associated with resistance exercise SBP independent of age and BMI (Koletsos et al., 2019) or resting and 24-h ambulatory blood pressure (Otsuki and Kotato, 2019).

Plasma NOx concentrations can represent NO derived from endothelial, neuronal, and inducible NO synthases and NOx from daily meals. However, we believe that the increases in plasma NOx concentrations observed in the training group reflect increases in NO production by endothelial and neuronal NO synthases. First, increases in plasma NOx concentrations were correlated with decreases in baPWV and exercise SBP. It has been reported that endothelial NO synthase mRNA expression is inversely correlated with arterial stiffness (Maeda et al., 2005) and that selective neuronal NO synthase blockade increases sympathetic blood pressure elevations during skeletal muscle contraction (Jendzjowsky et al., 2014). It is reasonable to consider that NO derived from endothelial or neural NO synthase decreases baPWV or exercise SBP, respectively. On the other hand, inflammation increases arterial stiffness (Vlachopoulos et al., 2005) and exercise blood pressure (Michishita et al., 2016). If the increases in plasma NOx concentrations were derived from inducible NO synthase, baPWV and exercise SBP would increase. Second, we asked subjects not to take dietary sources of NOx on the day before blood sampling. We checked their compliance to this instruction. Fujie et al. (2014, 2015) reported that increases in plasma NOx concentrations with aerobic training are correlated with changes in circulating levels of NO production–related substances, such as apelin and adropin. In addition, there is no evidence to suggest that NOx from daily meals is correlated with arterial stiffness and exercise SBP.

This study may have implications for exercise training in older individuals. First, the 6-week walking program induced 15-mmHg decreases in exercise SBP, although it might include familiarity with the exercise test. Schultz et al. (2013) reported that the relative risk of cardiovascular events increases 4% per 10-mmHg increase in SBP during aerobic exercise at moderate intensity. The training program may decrease SBP not only during the arm curl exercise but also during activities of daily living such as lifting objects, mopping, and climbing stairs and lead to lower cardiovascular risk. Second, resistance exercise SBP decreased with the training program without significant changes in resting SBP. This finding might be due to normotension at the time of enrollment, in accordance with a previous study (Otsuki et al., 2016). That previous study reported that levels of daily physical activity are correlated with resistance exercise SBP but not with resting SBP in healthy older adults (Otsuki et al., 2016). Resistance exercise SBP may be a more sensitive marker of the effects of aerobic exercise training than resting SBP.

This study has some limitations. First, the conclusions of this study were based on the stepwise regression results. However, statistical associations do not always imply causation. Further studies are needed to elucidate the role of NO in reducing resistance exercise SBP with aerobic training in older adults. Second, blood sampling was conducted only at rest; transient changes in NO production during resistance exercise were not investigated. Third, we could not investigate whether there are sex differences in resistance exercise SBP, plasma NOx concentrations, baPWV, and changes with the intervention in these measures because of the small sample size especially in men. In addition, factors other than NO might warrant investigation as mechanisms underlying the reduction in exercise SBP with aerobic training.

In conclusion, this study demonstrated that increases in plasma NOx concentrations with a 6-week aerobic exercise intervention are independently correlated with decreases in SBP during resistance exercise at 20% and 40% 1RM in older individuals. These results suggest that NO may be associated with decreases in resistance exercise SBP with aerobic training in older adults and help us better understand why habitual aerobic exercise prevents cardiovascular disease.

## Data Availability

The datasets generated for this study are available on request to the corresponding author.

## Ethics Statement

The studies involving human participants were reviewed and approved by the Ethics Committee of Ryutsu Keizai University (Approval Number 7). The patients/participants provided their written informed consent to participate in this study.

## Author Contributions

TO and AZ-M conceived and designed the research. TO, FN, and AZ-M performed the experiments and approved the final version of the manuscript. TO analyzed the data, prepared the figures, and drafted the manuscript. AZ-M and FN revised the manuscript.

## Conflict of Interest Statement

The authors declare that the research was conducted in the absence of any commercial or financial relationships that could be construed as a potential conflict of interest.

## References

[B1] Chaney R. H. Eyman R. K. (1988). Blood pressure at rest and during maximal dynamic and isometric exercise as predictors of systemic hypertension. *Am. J. Cardiol.* 62 1058–1061. 10.1016/0002-9149(88)90548-6 3189168

[B2] Chant B. Bakali M. Hinton T. Burchell A. E. Nightingale A. K. Paton J. F. R. (2018). Antihypertensive treatment fails to control blood pressure during exercise. *Hypertension* 72 102–109. 10.1161/HYPERTENSIONAHA.118.11076 29895532

[B3] Cohen J. (1992). A power primer. *Psychol. Bull.* 112 155–159. 19565683 10.1037//0033-2909.112.1.155

[B4] Dinenno F. A. Masuki S. Joyner M. J. (2005). Impaired modulation of sympathetic alpha-adrenergic vasoconstriction in contracting forearm muscle of ageing men. *J. Physiol.* 567 311–321. 10.1113/jphysiol.2005.087668 15946964 PMC1474179

[B5] Faul F. Erdfelder E. Lang A. G. Buchner A. (2007). G^∗^Power 3: a flexible statistical power analysis program for the social, behavioral, and biomedical sciences. *Behav. Res. Methods* 39 175–191. 10.3758/bf03193146 17695343

[B6] Fujie S. Hasegawa N. Sato K. Fujita S. Sanada K. Hamaoka T. (2015). Aerobic exercise training-induced changes in serum adropin level are associated with reduced arterial stiffness in middle-aged and older adults. *Am. J. Physiol. Heart Circ. Physiol.* 309 H1642–H1647. 10.1152/ajpheart.00338.2015 26371163

[B7] Fujie S. Sato K. Miyamoto-Mikami E. Hasegawa N. Fujita S. Sanada K. (2014). Reduction of arterial stiffness by exercise training is associated with increasing plasma apelin level in middle-aged and older adults. *PLoS One* 9:e93545. 10.1371/journal.pone.0093545 24691252 PMC3972107

[B8] Green L. C. Wagner D. A. Glogowski J. Skipper P. L. Wishnok J. S. Tannenbaum S. R. (1982). Analysis of nitrate, nitrite, and [15N]nitrate in biological fluids. *Anal. Biochem.* 126 131–138. 10.1016/0003-2697(82)90118-x 7181105

[B9] Himeno M. Ishibashi T. Nakano S. Furuya K. Kigoshi T. Uchida K. (2003). A practical procedure for achieving a steady state of NOx concentration in plasma: with special reference to the NOx content of Japanese daily food. *Tohoku J. Exp. Med.* 199 95–110. 10.1620/tjem.199.95 12705354

[B10] Jendzjowsky N. G. Delorey D. S. (2013). Short-term exercise training enhances functional sympatholysis through a nitric oxide-dependent mechanism. *J. Physiol.* 591 1535–1549. 10.1113/jphysiol.2012.238998 23297301 PMC3607171

[B11] Jendzjowsky N. G. Just T. P. DeLorey D. S. (2014). Exercise training augments neuronal nitric oxide synthase-mediated inhibition of sympathetic vasoconstriction in contracting skeletal muscle of rats. *J. Physiol.* 592 4789–4802. 10.1113/jphysiol.2014.278846 25194041 PMC4253477

[B12] Keller K. Stelzer K. Ostad M. A. Post F. (2017). Impact of exaggerated blood pressure response in normotensive individuals on future hypertension and prognosis: systematic review according to PRISMA guideline. *Adv. Med. Sci.* 62 317–329. 10.1016/j.advms.2016.11.010 28511070

[B13] Kim J. K. Kim K. A. Choi H. M. Park S. K. Stebbins C. L. (2018). Grape seed extract supplementation attenuates the blood pressure response to exercise in prehypertensive men. *J. Med. Food* 21 445–453. 10.1089/jmf.2017.0133 29683391

[B14] Koch D. W. Leuenberger U. A. Proctor D. N. (2003). Augmented leg vasoconstriction in dynamically exercising older men during acute sympathetic stimulation. *J. Physiol.* 551 337–344. 10.1113/jphysiol.2003.042747 12824451 PMC2343147

[B15] Kokkinos P. F. Narayan P. Fletcher R. D. Tsagadopoulos D. Papademetriou V. (1997). Effects of aerobic training on exaggerated blood pressure response to exercise in African-Americans with severe systemic hypertension treated with indapamide ± verapamil ± enalapril. *Am. J. Cardiol.* 79 1424–1426. 10.1016/s0002-9149(97)00158-6 9165176

[B16] Koletsos N. Dipla K. Triantafyllou A. Gkaliagkousi E. Sachpekidis V. Zafeiridis A. (2019). A brief submaximal isometric exercise test ‘unmasks’ systolic and diastolic masked hypertension. *J. Hypertens.* 37 710–719. 10.1097/HJH.0000000000001943 30817451

[B17] Kruse N. T. Hughes W. E. Hanada S. Ueda K. Bock J. M. Iwamoto E. (2018a). Evidence of a greater functional sympatholysis in habitually aerobic trained postmenopausal women. *J. Appl. Physiol.* 124 583–591. 10.1152/japplphysiol.00411.2017 28970201 PMC5899268

[B18] Kruse N. T. Ueda K. Hughes W. E. Casey D. P. (2018b). Eight weeks of nitrate supplementation improves blood flow and reduces the exaggerated pressor response during forearm exercise in peripheral artery disease. *Am. J. Physiol. Heart Circ. Physiol.* 315 H101–H108. 10.1152/ajpheart.00015.2018 29522355 PMC6087779

[B19] Maeda S. Iemitsu M. Miyauchi T. Kuno S. Matsuda M. Tanaka H. (2005). Aortic stiffness and aerobic exercise: mechanistic insight from microarray analyses. *Med. Sci. Sports Exerc.* 37 1710–1716. 10.1249/01.mss.0000175052.37087.f8 16260970

[B20] Maeda S. Tanabe T. Otsuki T. Sugawara J. Iemitsu M. Miyauchi T. (2004). Moderate regular exercise increases basal production of nitric oxide in elderly women. *Hypertens. Res.* 27 947–953. 10.1291/hypres.27.947 15894835

[B21] Michishita R. Ohta M. Ikeda M. Jiang Y. Yamato H. (2016). An exaggerated blood pressure response to exercise is associated with nitric oxide bioavailability and inflammatory markers in normotensive females. *Hypertens. Res.* 39 792–798. 10.1038/hr.2016.75 27334061

[B22] Michishita R. Ohta M. Ikeda M. Jiang Y. Yamato H. (2019). An exaggerated blood pressure response to exercise is associated with the dietary sodium, potassium, and antioxidant vitamin intake in normotensive subjects. *Clin. Exp. Hypertens.* 41 152–159. 10.1080/10641963.2018.1451539 29553836

[B23] Mizuno M. Iwamoto G. A. Vongpatanasin W. Mitchell J. H. Smith S. A. (2014). Exercise training improves functional sympatholysis in spontaneously hypertensive rats through a nitric oxide-dependent mechanism. *Am. J. Physiol. Heart Circ. Physiol.* 307 H242–H251. 10.1152/ajpheart.00103.2014 24816260 PMC4101645

[B24] Mortensen S. P. Nyberg M. Gliemann L. Thaning P. Saltin B. Hellsten Y. (2014). Exercise training modulates functional sympatholysis and alpha-adrenergic vasoconstrictor responsiveness in hypertensive and normotensive individuals. *J. Physiol.* 592 3063–3073. 10.1113/jphysiol.2014.273722 24860173 PMC4214660

[B25] Mortensen S. P. Nyberg M. Winding K. Saltin B. (2012). Lifelong physical activity preserves functional sympatholysis and purinergic signalling in the ageing human leg. *J. Physiol.* 590 6227–6236. 10.1113/jphysiol.2012.240093 22966164 PMC3530128

[B26] Nashar K. Nguyen J. P. Jesri A. Morrow J. D. Egan B. M. (2004). Angiotensin receptor blockade improves arterial distensibility and reduces exercise-induced pressor responses in obese hypertensive patients with the metabolic syndrome. *Am. J. Hypertens.* 17 477–482. 10.1016/j.amjhyper.2004.02.015 15177518

[B27] Otsuki T. Kotato T. (2019). Blood pressure during resistance exercise is associated with 24-h ambulatory blood pressure and arterial stiffness. *J. Phys. Fitness Sports Med.* 8 209–216. 10.7600/jpfsm.8.209

[B28] Otsuki T. Kotato T. Zempo-Miyaki A. (2016). Habitual exercise decreases systolic blood pressure during low-intensity resistance exercise in healthy middle-aged and older individuals. *Am. J. Physiol. Heart Circ. Physiol.* 311 H1024–H1030. 10.1152/ajpheart.00379.2016 27521421

[B29] Otsuki T. Maeda S. Iemitsu M. Saito Y. Tanimura Y. Ajisaka R. (2006). Contribution of systemic arterial compliance and systemic vascular resistance to effective arterial elastance changes during exercise in humans. *Acta Physiol.* 188 15–20. 10.1111/j.1748-1716.2006.01596.x 16911249

[B30] Otsuki T. Maeda S. Iemitsu M. Saito Y. Tanimura Y. Ajisaka R. (2008). Systemic arterial compliance, systemic vascular resistance, and effective arterial elastance during exercise in endurance-trained men. *Am. J. Physiol. Regul. Integr. Comp. Physiol.* 295 R228–R235. 10.1152/ajpregu.00009.2008 18463196

[B31] Otsuki T. Maeda S. Mukai J. Ohki M. Nakanishi M. Yoshikawa T. (2015a). Association between plasma sLOX-1 concentration and arterial stiffness in middle-aged and older individuals. *J. Clin. Biochem. Nutr.* 57 151–155. 10.3164/jcbn.15-27 26388674 PMC4566029

[B32] Otsuki T. Shimizu K. Maeda S. (2015b). Changes in arterial stiffness and nitric oxide production with *Chlorella*-derived multicomponent supplementation in middle-aged and older individuals. *J. Clin. Biochem. Nutr.* 57 228–232. 10.3164/jcbn.15-86 26566309 PMC4639594

[B33] Piche M. E. Laberge A. S. Brassard P. Arsenault B. J. Bertrand O. F. Despres J. P. (2018). Rosiglitazone lowers resting and blood pressure response to exercise in men with type 2 diabetes: a 1-year randomized study. *Diabetes Obes. Metab.* 20 1740–1750. 10.1111/dom.13293 29573098

[B34] Pitsavos C. Chrysohoou C. Koutroumbi M. Aggeli C. Kourlaba G. Panagiotakos D. (2011). The impact of moderate aerobic physical training on left ventricular mass, exercise capacity and blood pressure response during treadmill testing in borderline and mildly hypertensive males. *Hellenic J. Cardiol.* 52 6–14. 21292602

[B35] Schultz M. G. La Gerche A. Sharman J. E. (2017). Blood pressure response to exercise and cardiovascular disease. *Curr. Hypertens. Rep.* 19:89. 10.1007/s11906-017-0787-1 29046978

[B36] Schultz M. G. Otahal P. Cleland V. J. Blizzard L. Marwick T. H. Sharman J. E. (2013). Exercise-induced hypertension, cardiovascular events, and mortality in patients undergoing exercise stress testing: a systematic review and meta-analysis. *Am. J. Hypertens.* 26 357–366. 10.1093/ajh/hps053 23382486

[B37] Sugawara J. Komine H. Hayashi K. Yoshizawa M. Yokoi T. Otsuki T. (2007). Effect of systemic nitric oxide synthase inhibition on arterial stiffness in humans. *Hypertens. Res.* 30 411–415. 10.1291/hypres.30.411 17587753

[B38] Vlachopoulos C. Dima I. Aznaouridis K. Vasiliadou C. Ioakeimidis N. Aggeli C. (2005). Acute systemic inflammation increases arterial stiffness and decreases wave reflections in healthy individuals. *Circulation* 112 2193–2200. 10.1161/circulationaha.105.535435 16186422

[B39] Wang J. Brown M. A. Tam S. H. Chan M. C. Whitworth J. A. (1997). Effects of diet on measurement of nitric oxide metabolites. *Clin. Exp. Pharmacol. Physiol.* 24 418–420. 10.1111/j.1440-1681.1997.tb01212.x 9171946

[B40] Wilkinson I. B. MacCallum H. Cockcroft J. R. Webb D. J. (2002). Inhibition of basal nitric oxide synthesis increases aortic augmentation index and pulse wave velocity *in vivo*. *Br. J. Clin. Pharmacol.* 53 189–192. 10.1046/j.1365-2125.2002.1528adoc.x 11851643 PMC1874288

[B41] World Health Organization [WHO] (2018). *The Top 10 Causes of Death.* Available at: https://www.who.int/news-room/fact-sheets/detail/the-top-10-causes-of-death (accessed August 5, 2019).

